# Systematic review of factors associated with quality of life of asylum seekers and refugees in high-income countries

**DOI:** 10.1186/s13031-020-00292-y

**Published:** 2020-07-20

**Authors:** Catharina F. van der Boor, Rebekah Amos, Sarah Nevitt, Christopher Dowrick, Ross G. White

**Affiliations:** 1 Institute of Life and Human Sciences, University of Liverpool, Brownlow Hill, Liverpool, L69 3BX UK; 2 Institute of Translational Medicine, University of Liverpool, Crown Street, Liverpool, L69 3BX UK; 3 University of Liverpool, G.10, Ground floor, Whelan Building, Quadrangle, Brownlow Hill, Liverpool, L69 3GB UK

**Keywords:** Quality of life, Asylum seekers, Refugees, Migration

## Abstract

The stressful experiences that many asylum seekers and refugees (AS&R) are exposed to during forced migration, and during resettlement in host countries, can have a profound impact on their mental health. Comparatively less research attention has been allocated to exploring other indices of quality of life (QoL) in AS&R populations. This review aimed to (i) synthesize the predictors and correlates of QoL of AS&R populations in high-income countries, and (ii) to identify the methodological strengths and weaknesses of this body of research.

Fourteen databases were systematically searched (Medline, PsychINFO, CINAHL, Cochrane Library, Health Technology Assessment, National Health Service Economic Evaluation, Educational Resource Index and Abstracts, BiblioMap, Scopus, Social Sciences Citation Index, Evidence Aid, DARE, Web of Science and PubMed). Eligibility criteria included: adults seeking asylum or refuge in a high-income country, primary quantitative data, the use of a measure based on the WHO’s definition of QoL, published in a peer-reviewed journal. A narrative synthesis approach was used, and the quality was assessed using the AXIS tool for cross-sectional studies and the CASP tool for longitudinal studies.

Of the 13.656 papers identified, 23 met the eligibility criteria. A wide range of factors were found to have significant associations with QoL. Both positive and negative correlates of QoL were largely dominated by social (e.g. social networks) and mental health factors (e.g. depression). Although all of the cross-sectional studies met over half of the quality criteria, only 12 met 75% or more of these criteria. For the longitudinal studies, for all but one study lacked statistical precision and the results cannot be applied to the local population.

Key findings across the various forms of QoL (overall, physical, psychological, social and environmental) were that having established social networks and social integration were associated with higher QoL, whereas having mental disorders (i.e. PTSD or depression) was strongly associated with reduced QoL. More research is needed into physical and environmental predictors and correlates of QoL. The findings of the review can be used to inform policies and interventions aimed at supporting AS&R and promoting the integration and wellbeing of these populations.

## Introduction

The number of forcibly displaced persons in 2018 exceeded 70.8 million worldwide [1]. Within this displaced group, the estimated number of people awaiting a decision on their application for asylum was 3.5 million, and an estimated 25.9 million individuals were recognized as refugees [1]. High income countries on average host 2.7 refugees per 1000 of population [1]. The stressful experiences that many asylum seekers and refugees (AS&R) are exposed to during forced migration, and during resettlement in host countries, can have a profound impact on their mental health (MH) including high rates of depression, anxiety and posttraumatic stress disorder [2]. However, comparatively less research attention has been allocated to exploring other indices of MH such as quality of life (QoL) in AS&R populations.

### Quality of life

QoL has been implicated in MH status. It is defined as an ‘Individuals’ perception of their position in life in the context of the culture and value systems in which they live and in relation to their goals, expectations, standards and concerns’ ([3], p.1). As such, QoL is a broad ranging and multidimensional concept which includes an individual’s subjective evaluation of their physical health, psychological state, level of independence, social relationships, personal beliefs and their relationship to their environment [4].

Whilst there is growing consensus over the multidimensionality of QoL, little research has focused on understanding the specific predictors and correlates thereof. This is specifically the case with regards to AS&R populations, despite the existing evidence base for their high risk of developing mental disorders. The WHO estimates the prevalence of mental disorders, including depression, anxiety, post-traumatic stress disorder (PTSD), bipolar disorder and schizophrenia, in conflict-affected settings to be 22.1% at any time point in the populations assessed [5]. Evidence has shown that for AS&R the effects of war-related events may persist for years and have been associated with lower QoL even when hostilities have ended [6, 7]. Akinyemi et al. [6] noted that QoL, together with occupational status, were the biggest threats to the mental health of refugee populations and called for attention to the overall QoL in order to support their long-term mental health. Similarly, Matanov et al. [7], found that traumatic war events were directly associated with lower QoL in war-affected communities in the Balkan countries, and experiencing more migration-related stressors was linked to lower QoL in refugee populations who had resettled in Western Europe. Simultaneously, the lack of studies evaluating the efficacy of interventions for increasing QoL in AS&R populations [8, 9] has been noted. Improving understanding about predictors and correlates of QoL in AS&R populations will be important for guiding the foci of these interventions, and more broadly informing policies in high-income countries to support the local settlement, integration and long-term mental health of AS&Rs.

The current paper is the first to systematically review evidence relating to predictors and correlates of QoL of AS&Rs living in high-income countries. The specific aims of the review were to: 1) understand what factors are associated with QoL in AS&R populations; 2) identify the methodological strengths and weaknesses of the research investigating QoL.

## Methods

### Literature search

Fourteen databases were systematically searched. A search strategy tailored to the aims of the review was applied to each database using the Kings College London library guide [10]. See appendix A for the list of databases which were searched and the full search strategy. Reference chaining was also carried out and five experts in the field of mental health of refugee populations were independently consulted to ensure the final list of included papers was exhaustive.

### Eligibility

All quantitative peer-reviewed publications in English, Spanish or Dutch (languages spoken by the authors of this review) which used measures based on the four WHOQOL domains [4], explored predictors and correlates of the QoL of adult AS&R populations residing in a high-income country (as classified by the World Bank^1^) at the time that the search was conducted were included. The exclusion of grey literature was used as a form of minimal quality assurance. Longitudinal evaluations of interventions were also excluded if a cross-sectional analysis between QoL and other variables were not performed at baseline. The search of databases was conducted up to the 5th of May 2020, and any studies that met inclusion criteria were included in the current review. Furthermore, additional papers identified through expert consultation were included.

CB and RA independently screened the titles and abstracts for inclusion. Articles rated as possible candidates by either CB or RA were added to a preliminary list. Working independently and in duplicate, both reviewers inspected the full texts of the preliminary list for inclusion. A consensus meeting was subsequently held between CB and RA and remaining discrepancies were resolved through discussion with the research team.

### Data extraction and quality appraisal

For each included study, CB extracted information on the publication year, country of publication, settings, populations, study design, assessment measures and key findings, which was peer-reviewed by RW. Once the data was extracted, CB rated the quality of each individual study and RA peer reviewed the quality appraisal for a quarter of the studies.

### Data synthesis and analysis

A narrative synthesis approach was used to analyze the data. The WHOQOL Group developed a conceptual framework for QoL that incorporates four domains [4]: physical, psychological, social relationships and environment. To support efforts to synthesize the research findings of the studies included in the current review, these four domains were used to group predictors and correlates of QoL investigated in the studies. Two authors (CB and RW) independently mapped the various correlates investigated in the studies onto these four domains, discrepancies were resolved through discussion.

Consideration was given to conducting a meta-analysis. Five studies reported significant relationship between QoL and the correlates in terms of a correlation coefficient (an r statistic) and the statistical significance of this coefficient. Meta-analysis of correlation coefficients is methodologically complex due to the bounded nature of these statistics (i.e. that they can only take values between: − 1 and + 1) and furthermore, correlation coefficients were not reported with a measure of precision such as a confidence interval which would be required for meta-analysis [11, 12]. Similarly, five studies also reported the relationship between QoL and the variables in terms of t-statistics and corresponding *p*-values. Such statistics do not have an associated measure of precision and therefore cannot be combined within meta-analysis.

The only amenable statistical measure for meta-analysis to represent the relationship between QoL and the predictors were regression (beta) coefficients with accompanying confidence intervals. These were reported in 52% of studies. These act as continuous data and theoretically, synthesis of such data may be possible within a ‘prognostic review’ framework [13]. However, across the studies, the predictors included within regression models to examine the effects of these predictors on QoL varied widely. Therefore, due to anticipated very large heterogeneity originating from the wide range of predictors included within regression models (see Additional file 1), meta-analysis of these beta-coefficients was deemed to be potentially misleading and therefore inappropriate.

Instead, further consideration of the direction, strength and consistency of the correlates of overall QoL has been undertaken for the studies included in this review which reported correlational analysis. Cohen’s [14] conventions were used to interpret the effect sizes; positive large correlation (> 0.50 to + 1.00), positive moderate correlation (0.30 to 0.50) and positive small correlation (< 0.30). Negative large correlation (<− 0.50 to − 1.00), negative moderate correlation (− 0.30 to 0.50) and negative small correlation (> − 0.30). Positive correlations indicate a relationship between two variables in which both variables move in the same direction (i.e. if mental health increases, QoL increases), whereas negative correlations indicate a relationship whereby both variables move in opposite directions (i.e. if depression decreases, QoL increases). Figure 2 provides a representation thereof.

The quality of the cross-sectional studies was assessed using the *Appraisal tool for Cross-Sectional Studies* (AXIS tool) [15]. Longitudinal studies were assessed using the *Critical Appraisal Skills Programme* [16]. The quality of the studies was independently rated by CB. Additionally, RA rated a quarter of the cross-sectional studies (*N* = 5) and longitudinal studies (*N* = 2) in order to ensure a quality check was carried out. There was high agreement regarding quality assessment; items that were rated differently were resolved through discussion.

## Results

**Fig. 1:**
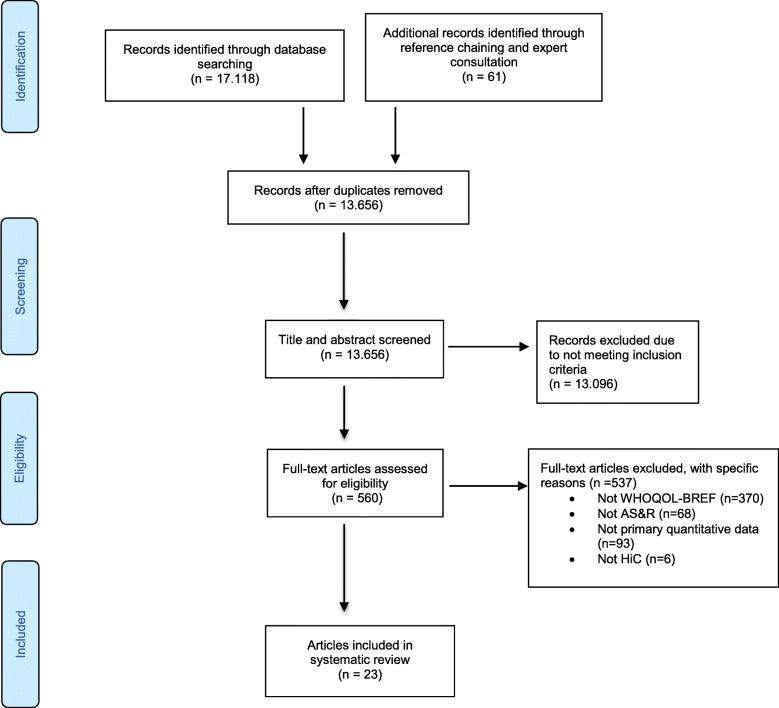
PRISMA flow diagram of article selection

The search identified 13.655 articles of which 23 met the inclusion criteria. Article selection is summarized in the PRISMA diagram presented in Fig. 1.

### Study characteristics

A total of twenty-three studies met the inclusion criteria. Seventeen studies were conducted in Europe, two in Australia, two in Israel, one in the USA and one in Japan. Four studies used the same dataset, therefore there were two repeat samples. Sample sizes ranged from 22 to 663 (Mdn = 119, IQR = 222), with a total sample of 3817 across studies including 2138 males, 1516 females, and 163 not specified. Studies that used the same dataset were only counted once. Eleven of the studies recruited individuals from a medical setting, and the rest were recruited from support agencies, reception facilities (*N* = 5), community events (*N* = 4) or other (*N* = 3). Seventeen studies reported cross-sectional data and six were longitudinal studies including one case control design. All studies used the WHOQOL-100, WHOQOL-BREF or the EUROHIS-QOL measures (see Table 1).

**Table 1:** Summary table of the selected articles including study site, country of origin, sample size, type of migrant, study design, assessment tool and findings

Study	Country in which the Study was Conducted	Migrant Country of Origin	N	Type of Migrant	Study Design	Recruitment Site	Self-Rating Scale for QoL	Other validated Assessment Measures	Summary of Significant Associations with QoL	Non-significant Associations with QoL (***p*** > .005)
Carlsson et al. 2010^a^ [17]	Denmark	Iraq, Iran, Afghanistan	45	Refugees	Longitudinal	Rehabilitation and research center for torture victims	WHOQOL-Bref (WHOQOL Group, 1998)	HTQ, HSCL-25, HDS	● WHOQOL Environment Time (Baseline vs. 9-month follow-up)(*p* = .029) ● WHOQOL Environment Time (Baseline vs. 23-month follow-up)(*p* = .017)	• No significant difference between baseline and 9 month, or 23 month follow-up for WHOQOL Physical, mental or social.
Carlsson, Mortensen & Kastrup^a^ [18]	Denmark	Iraq, Iran, Afghanistan	55	Refugees	Longitudinal	Rehabilitation and research center for torture victims	WHOQOL-Bref (WHOQOL Group, 1998) [4]	HTQ, HSCL-25, HDS	● Changes in mental health ○ Evaluation of improved mental health (vs. those evaluating no improvement) during treatment had higher health-related quality of life in the ‘mental’ domain (*t* = 2.46, *p* = .017) ● Those with the lowest baseline QoL showed the largest increase in QoL.	• No significant changes over time for the WHOQOL domains. • The Spearman rank correlations between years from exposure to torture and baseline scores were nonsignificant for all QoL domains • The Spearman rank correlation between total number of treatment sessions and difference scores was low and nonsignificant for all QoL domains • Expressing expectations to improve during treatment was not associated with changes in QoL domains
Carlsson, Mortensen & Kastrup [19]	Denmark	Iraq, Iran, Afghanistan, other (not specified)	63	Refugees	Cross-Sectional	Rehabilitation and research center for torture victims	WHOQOL-Bref [4]	HSCL-25, HDS, HTQ	● Overall variance accounted for by the regression model was not reported ● WHOQOL Physical ○ Occupation (*β* = 0.23, *p* < .05) ○ Social relations (*β* = 0.33, *p* < .01) ○ Pain (*β* = − 0.42, *p* < .01) ● WHOQOL psychological ○ Social relations (*β* = 0.31, *p* < .05) ● WHOQOL Social ○ Social relations (*β* = 0.39, *p* < .01) ● WHOQOL Environment ○ Social relations (*β* = 0.40, *p* < .01) ▫ Pain (*β* = − 0.35, *p* < .05)	o Number of years since last exposure to torture was not associated with QoL o Age and proficiency in Danish were not significant associated with QoL Regression (Model 2) • Education, torture, or having been on the run were not significantly associated with any of the QoL domains • Occupation was not significantly associated with mental, social or environmental QoL • Pain was not significantly associated with mental or social QoL
Carlsson, Olsen, Mortensen & Kastrup* [20]	Denmark	Iran, Iraq, Lebanon	139	Refugees	Longitudinal	Rehabilitation and research center for torture victims	WHOQOL-Bref [4]	HTQ, HSCL-25	● Overall variance accounted for by the regression model was not reported ● WHOQOL Physical ○ Pain upper extremities (*β* = 0.20, *p* < .05) ○ Employment (*β* = 0.49, *p* < .001) ▫ Headache (*β* = − 0.31, *p* < .001) ● WHOQOL Mental ○ Social relations (*β* = 0.21, *p* < .05) ○ Employment (*β* = 0.40, *p* < .001) ● WHOQOL Social ○ Social relations (*β* = 0.40, *p* < .001) ○ Employment (*β* = 0.27, *p* < .01) ▫ Headache (*β* = − 0.20, *p* < .05) ● WHOQOL Environment ○ Social relations (*β* = 0.24, *p* < .01) ○ Employment (*β* = 0.36, *p* < .001) ▫ Headache (*β* = − 0.19, *p* < .05)	Regression (Model 2) • Education, marked mood shifts, and years in Denmark were not associated with any of the QoL domains • Pain in upper extremities was not associated with mental, social or environmental QoL • Headache was not associated with mental QoL • Social relations was not associated with physical QoL
Correa-Velez, Green, Murray [21]	Australia	Africa, South Asia, Middle East, West Asia, South East Asia	104	Refugees	Cross-Sectional	Agency involved in refugee resettlement	WHOQOL-Bref	HTQ, PMLD, SASCAT	● Regressing WHOQOL Physical domain on predictor variables was significant (*r*^2^ = .30, *p* < .001) ○ Region of birth (Africa) (*β* = 0.32, 95% CI = [5.44, 19.83], *p* = .001) ○ Education (2y/3y) (*β* = 0.32, 95% CI = [3.80, 16.94], *p* = .002) ○ Community can be trusted (*β* = 0.20, 95% CI = [.79, 12.49], *p* = .027) ● Regressing WHOQOL Psychological domain on predictor variables was significant (*r*^2^ = .19, *p* = .008) ○ Community can be trusted (*β* = 0.20, 95% CI = [.33, 13.02], *p* = .039) ▫ Number of people no support (*β* = − 0.24, 95% CI = [− 5.80, −.57], *p* = .018)	• Age, Children (1 or more), English skills and trauma types were non-significant predictors of the physical domain • Age, region of birth (Africa), children (1 or more), education (2y/3y), English skills, and trauma types were non-significant predictors of the psychological domain • The hierarchical logistic regression predicting overall QOL found no significant associations
Correa-Velez, Barnett, Gifford & Sackey* [22]	Australia	Sudan, Burma (Myanmar), Iraq, Burundi, the Democratic Republic of Congo, Rwanda, Liberia, Afghanistan, Congo-Brazzaville, Iran, Tanzania, Uganda	233	Refugees	Cross-Sectional	Community	WHOQOL-Bref [4]	HSCL-25, HTQ, Items to assess use of Health Services & Medication	● WHOQOL Environment ▫ Living in regional areas (OR = 0.4, 95% CI = [0.2, 0.9], *p* < .05)	• Area of settlement did not predict significant poorer QoL in the physical, mental or social domain
Georgiadeu et al. [23]	Germany	Syrian	119	Refugees	Cross-sectional	Registry	WHOQOL-Bref	ETI, PHQ-9, GAD-7, SOC-13, F-SozU,	● WHOQOL Psychological Married with partner in Germany scored higher than married without partner in Germany, t(117) = 2.91, *p* = .004 ● WHOQOL Social Married with partner in Germany scored higher than married without partner in Germany, U = -3.02, *p* = .002 ● WHOQOL Environment Married with partner in Germany scored higher than married without partner in Germany, t(117) = 2.27, *p* = .025. ● WHOQOL Overall Married with partner in Germany scored higher than married without partner in Germany, t(117) = 2.78, *p* = .006. ● Regressing overall QoL on predictor variables was significant (*r*^2^ = .66) ○ Sense of coherence (*β* = 0.15, 95% CI = [− 0.00, 0.33], *p* = .049) ○ Social support (*β* = 0.25, 95% CI = [0.15, 0.46], *p* < .001) ▫ Depression (*β* = − 0.44, 95% CI = [− 1.52, − 0.61], *p* < .001) ● Regressing WHOQOL psychological domain on predictor variables was significant (*r*^2^ = .61) ○ Gender (*β* = 0.15, 95% CI = [0.32, 11.04], *p* = .038) ○ Residence of partner (*β* = 0.17, 95% CI = [1.39, 13.95], *p* = .017) ○ Sense of coherence (*β* = 0.22, 95% CI = [0.07, 0.47], *p* = .008) ○ Social support (*β* = 0.17, 95% CI = [0.04, 0.41], *p* = .016) ▫ Depression (*β* = − 0.40, 95% CI = [− 1.66, − 0.56], *p* < .001)	• No significant differences in WHOQOL Physical (married with partner vs. married without partner) • Sex, age, residence of partner, residence of minor child, anxiety, number of traumatic events, trauma inventory, and satisfaction with marriage were non-significant predictors of overall QoL • Age accommodation, residence of minor child, anxiety, number of traumatic events, trauma inventory, and satisfaction with marriage were non-significant predictors of WHOQOL psychological.
Ghazinour, Richter & Eisemann [24]	Sweden	Iran	100	Refugees	Cross-Sectional	Half were recruited as outpatients at a psychiatric clinic and half were recruited as interested volunteers.	WHOQOL-100 [18]	CRI, ISSI, BDI, SCL-90	● Gender: Males reported lower overall QoL (t = − 2.99, *p* = .004) than females ● Males reported lower levels of Independence (Psychological domain), (t = − 2.00, *p* = .049) than females ● Males reported lower social QoL than females (t = − 2.40, *p* = .018) ● Males reported lower environmental QoL (t = − 2.06, *p* = .043) ● Males reported lower spirituality (psychological domain) (*t = − 2.82, p = .006*) ● Having a BDI score below the mean and having been in the army showed the highest significant overall QoL F(19,70) = 60.06, *p* < .001 ● Sense of coherence, coping resources, and social support had various significant relationships with QoL (see paper for details)	• Gender: no significant differences found for physical health or psychological health • No significant correlation was found between spirituality (psychological domain) and adequacy of attachment (social support scale)
Hengst, Smid & Laban^b^ [25]	Netherlands	Iraq	294	Asylum Seekers	Cross-Sectional	Central Organ of Asylum (COA)	WHOQOL-Bref [4]	HTQ, PMLP, WHO-CIDI, BDQ	● Mediation model of psychopathology, disability and quality of life χ^2^(12) = 10.52, *p* = .570 ▫ Unnatural loss of a child (*β* = −.05, 95% CI = [−.44, −.03], *p* < .05) ▫ N° of lost family members (*β* = −.04, 95% CI = [−.01, .00], *p* < .05) ▫ Other traumatic events (*β* = −.13, 95% CI = [−.06, −.02], *p* < .05) ▫ Long asylum procedure (*β* = −.18, 95% CI = [−.66, −.13], *p* < .05) ▫ Psychopathology (*β* = −.33, 95% CI = [−.42, −.20], *p* < .05)	• Age, female sex, education level and postmigration stressors were not significantly associated with QoL • Unnatural loss of family, unnatural loss of friends, witnessing the loss of family or friend, number of lost children, and number of lost friends were not significantly associated with QoL
Huijts, Kleijn, van Emmerik, Noordhof, and Smith [26]	Netherlands	38 different countries in the Middle East, former Yugoslavia, or other regions of which 50 were Asian, 35 African, and 4 South American.	335	Refugees	Cross-Sectional	Foundation Centrum ‘45, a specialist institute for diagnosis and treatment of posttraumatic stress.	WHOQOL-Bref [4]	COPE-EASY-32, HTQ	● Regressing overall QoL on predictor variables was significant (*r*^2^ = .42, *p* < .05) ○ Social Support Seeking (*β* = 0.12, 95% CI = [.03, .21], *p* < .05) ○ Emotion-Focused Coping (*β* = 0.13, 95% CI = [.04, .23], *p* < .01) ○ Self-reported PTSD (*β* = − 0.61, 95% CI = [−.68, −.54], *p* < .001) ● Post-hoc analyses revealed that emotion-focused coping and social support seeking differed per country of origin, and per gender.	Subgroup analysis of regression o Males: emotion-focused coping, was not significantly related to QoL o Females: social support seeking was not significantly related to QoL Multigroup analyses • No significant differences found regarding length of stay in the Netherlands.
Jesuthasan et al.* [27]	Germany	Afghanistan, Syria, Iraq, Somalia, Iran, Eritrea	663	Refugees + European Reference Sample	Cross-Sectional	Shared reception facilities	EUROHIS-QOL questionnaire [24]	HTQ, HSCL-25, ICSEY	● Female refugees rated their overall QoL significantly lower than the EU reference simple, t(5508) = 16.9, *p* < .0001 ● Residence and mission in a war zone, and being sick without any access to health care significantly affected all four domains of QoL ● Near death experience affected physical and psychological domains. ● Aggression from family members affected the physical and social domain ● Forced isolation affected the physical, psychological, and environmental domains. ● Within Group (Refugees) Predictors: Regressing overall reduced QoL on socio-demographic and traumatic predictor variables (Overall variance accounted for by the regression model was not reported) A) Reduced QoL ● Age > 30 (OR = 1.6, 95% CI = [1.2–2.3], *p* = .004) ● Near-Death Experience (OR = 1.7, 95% CI = [1.2, 2.4], *p* = .001) ● Mission/Residence in War Zone (OR = 0.7, 95% CI = [0.5–1.0], *p* = .04) ● Attack by Family Member (OR = 2, 95% CI = [1.3, 3.1], *p* = .001)	• Having had sexual contacts as a minor did not signfiicantly correlate with overall QoL • No significant association was found between near death experience and the social and environmental domains • No significant association was found between aggression from family members and the psychological and environmental domains • No signiciant association was found between forced isolation and the social domain.
Kinzie et al. [28]	USA	Ethiopia, Somalia, Iran and Afghanistan	22	Refugees	Longitudinal	Refugee psychiatric clinic	WHOQOL-BREF	HTQ, SDS, CES-D	● WHOQOL Physical ○ Time (baseline vs. 1-year follow-up) (*p* < .001) ● WHOQOL Psychological ○ Time (baseline vs. 1-year follow-up) (*p* < .001) ● WHOQOL Environment ○ Time (baseline vs. 1-year follow-up) (*p* = .004)	None reported
Laban, Gernaat, Komproe & de Jong^b^ [29]	Netherlands	Iraq	294	Asylum Seekers *Group 1*: living in the Netherlands < 6 months *Group 2*: living in the Netherlands for at least 2 years.	Cross-Sectional	Agency for the reception of asylum seekers	WHOQOL-Bref [4]	PMLP, WHO-CIDI, Physical Health Rating	● Overall QoL group 1 vs. group 2 (*p* < .0005, Z(294) = − 5.29) with group 2 scoring lower than group 1 ● Perceived QoL General Health group 1 vs. group 2 (*p* = .017, Z(294) = − 2.39) with group 2 scoring lower than group 1.	None reported
Laban, Komproe, Gernaat & de Jong^b^ [30]	Netherlands	Iraq	294	Asylum Seekers *Group 1*: living in the Netherlands < 6 months *Group 2*: living in the Netherlands for at least 2 years.	Cross-Sectional	Agency for the reception of asylum seekers	WHOQOL-Bref [4]	HTQ, PMLP, WHO-CIDI	● Overall QoL was significantly lower in group 2 Z(294) = − 5.29, *p* = .0005 ● WHOQOL physical was significantly lower in group 2, t(292) = 3.21, *p* = .001 ● WHOQOL psychological was significantly lower in group 2, t(292) = 2.33, *p* = .020 ● WHOQOL environment was significantly lower in group 2, t(292) = 5.26, *p* = .001 ● Regressing overall QoL on predictor variables was significant (*r*^2^ = 0.13, *p* < .001) ▫ Long Asylum Procedure (*β* = − 0.17, *p* < .01) ▫ Adverse life events after arrival in the Netherlands (*β* = − 0.13, *p* < .05) ▫ WHOQOL Physical (*r*^2^ = .31, *p < .*01) ▫ Adverse life events after arrival (*β* = − 0.15, *p* < .05) ▫ Depression (*β* = − 0.19, *p* < .01) ▫ Somatoform disorders (*β* = − 0.12, *p* < .05) ▫ One or more psychiatric disorders (*β* = − 0.19, *p* < .05) ▫ Older age (*β* = − 0.14, *p* < .01) ▫ Socio-economic living conditions (*β* = − 0.20, *p* < .01) ● WHOQOL Psychological (*r*^2^ = .18, *p < .*01) ▫ Self-reported PTSD (*β* = − 0.17, *p* < .05) ▫ Somatoform disorders (*β* = − 0.15, *p* < .01) ▫ Socio-economic living conditions (*β* = − 0.14, *p* < .05) ▫ Anxiety (*β* = − 0.17, *p* < .05) ● WHOQOL Social (*r*^2^ = .12, *p < .*01) ● WHOQOL Environmental (*r*^2^ = .15, *p < .*01) ○ Socio-religious aspects (*β* = 0.12, *p* < .05) ▫ Self-rated PTSD (*β* = − 0.14, *p* < .05) ▫ Socio-economic living conditions (*β* = − 0.27, *p* < .01)	• WHOQOL social was not significantly different between group 1 and group 2. Regression • Psychopathology and socio-economic living conditions were not associated with overall QoL • Anxiety disorders, PTSD, long asylum procedure, adverse life events after arrival, and family issues were not associated with physical QoL • Having one or more psychiatric disorders, depressive disorders, a long asylum procedure, adverse events after arrival and family issues were not associated with psychological QoL • Psychopathology, adverse events after arrival, family issues and socioeconomic living conditions were not associated with social QoL • One or more psychiatric disorders, depressive disorders, anxiety disorders, somatoform disorders, long asylum procedure, adverse events after arrival, and family issues were not associated with environmental QoL
Lee et al. [31]	Japan	North Korea	81	Refugees (resettled in Japan vs. resettled in South Korea)	Cross -sectional	Support center	WHOQOL-Bref	BDI	● Resettled in Japan vs. Resettled in South Korea ● Overall QOL (*p* < .05), Korea scoring higher ● WHOQOL Physical (*p* < .05), Korea scoring higher ● WHOQOL Mental (*p* < .01), Korea scoring higher ● WHOQOL social (*p* < .05), Korea scoring higher ● WHOQOL environment (*p* < .001), Korea scoring higher	None reported
Leiler et al. [32]	Sweden	Afghanistan, Syria, Iraq, Iran, Eritrea, Somalia	510	AS&R	Cross-sectional	Housing facilities	WHOQOL-BREF	PHQ-9, GAD-7, PC-PTSD	● WHOQOL Physical ▫ Depression (*r* = − 0.58, *p* < .001) ▫ Anxiety (*r* = − 0.52, *p* < .001) ▫ PTSD (*r* = − 0.36, *p* < .001) ● WHOQOL Psychological ▫ Depression (*r* = − 0.38, *p* < .001) ▫ Anxiety (*r* = − 0.32, *p* < .001) ▫ PTSD (*r* = − 0.21, *p* < .001) ● WHOQOL Social ▫ Depression (*r* = − 0.37, *p* < .001) ▫ Anxiety (*r* = − 0.37, *p* < .001) ▫ PTSD (*r* = − 0.27, *p* < .001) ● WHOQOL Environment ▫ Depression (*r* = − 0.34, *p* < .001) ▫ Anxiety (*r* = − 0.33, *p* < .001) ▫ PTSD (*r* = − 0.23, *p* < .001)	No significant differences found between asylum seekers and refugees neither in the domain scores nor in overall QoL score.
Löfvander, Rosenblad, Wiklund, Bennström & Leppert [33]	Sweden	Somalia, Iraq, Syria	66 pairs of refugees and matched Swedish born	Refugees	Longitudinal Case-Control	Asylum and integration healthcare center	WHOQOL-Bref [4]	GHQ-12, GAF	● Between Groups (Men) ○ Psychological (Baseline; *p* = .020) ○ Social Relations (Baseline; *p* = .002, 6 Months *p* < .001, 12 Months *p* = .001) ● Between Groups (Women) ○ Social Relations (6 Months; *p* = .030) ● Between Groups (Mixed) ○ Psychological (Baseline; *p* = .004, 6 Months; *p* = .025, 12 Months; *p* = .041) ○ Social (Baseline; *p* = .002, 6 Months; *p* < .001, 12 Months; *p* = .001)	Between groups (men) • No significant differences for physical QoL or environmental QoL at any timepoint. • No significant differences at 6-months or 12-months for psychological QoL. Between groups (women) • No significant differences for physical, psychological or environmental QoL at any timepoint. • No significant differences at baseline or at 12 months for social QoL Between groups (mixed) • No significant differences for physical or environmental QoL at any timepoint
Regev & Slonim-Nevo [34]	Israel	Sudan	300	AS&R	Cross-sectional	Community	WHOQOL-Bref	HTQ, PCL-C, BSI, MSPSS	● Overall model for WHOQOL was significant (*r*^2^ = 0.07, *p* < .001) ○ Social support *(β* = 0.32, *p* < .001) ○ Other’s traumatic events *(β* = 0.27, *p* < .001) ▫ Gender *(β* = − 0.32, *p* < .001) ▫ Self-traumatic events *(β* = − 0.20, *p* < .001)	● Length of stay was not a significant predictor of QoL
Slonim-Nevo [35]	Israel	Sudan	340	AS&R	Cross-sectional	Community	WHOQOL-Bref	HTQ, Language proficiency in Hebrew, PMLD, perceived discrimination, PCL-C, BSI, AIS, CSQ, FAD, MSPSS	● Overall model for WHOQOL Physical was significant (*r*^2^ = 0.32, *p* < .001) ○ Legal status *(β* = 0.14, *p* < .01) ▫ PTSD *(β* = − 0.40, *p* < .001) ▫ Perceived discrimination *(β* = − 0.30, *p* < .001) ● Overall model for WHOQOL Psychological was significant (*r*^2^ = 0.31, *p* < .001) ▫ PTSD status *(β* = − 0.29, *p* < .001) ▫ Perceived discrimination *(β* = − 0.38, *p* < .001) ● Overall model for WHOQOL Social was significant (*r*^2^ = 0.12, *p* < .001) ▫ PTSD *(β* = − 0.27, *p* < .001) ▫ Perceived discrimination *(β* = − 0.15, *p* < .05) ● Overall model for WHOQOL Environment was significant (*r*^2^ = 0.25, *p* < .001) ▫ Perceived discrimination *(β* = − 0.24, *p* < .001) ▫ Post-migration living difficulties *(β* = − 0.38, *p* < .001)	● WHOQOL Physical ○ Gender ○ Post-migration living difficulties ● WHOQOL psychological ○ Gender ○ Legal status ○ Post-migration living difficulties ● WHOQOL Social ○ Gender ○ Legal status ○ Post-migration living difficulties ● WHOQOL Environment ○ Gender ○ Legal status ○ PTSD diagnosis
Stammel et al. [36]	Germany	Iran, Chechnya, Turkey, Syria, Kosovo, Afghanistan, Iraq, Other countries of the russian Federation, Armenia, Kenya, Angola, Chile, Lebanon	76	AS&R	Longitudinal	Center for torture victims	EUROHIS-QOL	MINI, PDS, HSCL-25, SCL-90-R	● Multilevel analysis revealed QoL increased significantly after an average of 14 months of treatment (Pseudo *R*^2^ = .14, *β* = 0.42, 95% CI [0.29, 0.55], *p* < .001).	● Not specified
Teodorescu, Siqveland, Heir, Hauff, Wentzel-Larsen & Lien [37]	Norway	Eastern Europe, Africa, Middle East, Far East, Latin America	55	Refugees	Cross-Sectional	Hospital outpatient department	WHOQOL-Bref [4]	LEC, CAPS, SCID-PTSD, MINI, IES-R, HSCL-25, PTGI-SF	● Bivariate correlations ● WHOQOL Physical ○ Posttraumatic growth (r_s_ = .51, *p* < ..001) ▫ Weak social network (r_s_ = −.35, *p* < .01) ▫ Poor social integration (r_s_ = −.32, *p* < .05) ▫ Unemployment (r_s_ = −.34, *p* < .05) ▫ Posttraumatic stress (rs = −.45, *p* < .01) ▫ Depression (*r*_s_ = −.59, *p* < .001) ● WHOQOL Psychological ○ Posttraumatic growth (r_s_ = .58, *p* < ..001) ○ Physical QoL (r_s_ = .73, *p* < .001) ▫ Weak social network (r_s_ = −.53, *p* < .001) ▫ Poor social integration (r_s_ = −.37, *p* < .01) ▫ Unemployment (r_s_ = −.31, *p* < .05) ▫ Posttraumatic stress (r_s_ = −.53, *p* < .001) ▫ Depression (r_s_ = −.58, *p* < .001) ● WHOQOL social ○ Posttraumatic growth (r_s_ = .41, *p* < .01) ○ Physical QoL (r_s_ = .46, *p* < .001) ○ Psychological QoL (r_s_ = .54, *p* < .001) ▫ Weak social network (r_s_ = −.61, *p* < .001) ▫ Poor social integration (r_s_ = −.48, *p* < .001) ▫ Unemployment (r_s_ = −.37, *p* < .01) ▫ Posttraumatic stress (r_s_ = −.45, *p* < .01) ▫ Depression (r_s_ = −.54, *p* < .001) ● WHOQOL environment ○ Posttraumatic growth (r_s_ = .49, *p* < .001) ○ Physical QoL (r_s_ = .48, *p* < .001) ○ Psychological QoL (r_s_ = .53, *p* < .001) ○ Social QoL (r_s_ = .62, *p* < .001) ▫ Weak social network (r_s_ = −.56, *p* < .001) ▫ Poor social integration (r_s_ = −.40, *p* < .01) ▫ Unemployment (r_s_ = −.38, *p* < .01) ▫ Posttraumatic stress (r_s_ = −.38, *p* < .01) ▫ Depression (rs = −.51, *p* < .001) ● Overall QoL ○ Posttraumatic growth (r_s_ = .47, *p* < .001) ○ Physical QoL (r_s_ = .62, *p* < .001) ○ Psychological QoL (r_s_ = .71, *p* < .001) ○ Social QoL (r_s_ = .39, *p* < .01 ○ Environmental QoL (r_s_ = .48, *p* < .001) ▫ Weak social network (r_s_ = −.39, *p* < .01) ▫ Poor social Integration (r_s_ = −.38, *p* < .01) ▫ Posttraumatic stress (r_s_ = −.65, *p* < .001) ▫ Depression (r_s_ = −.70, *p* < .001) ● Regression ● WHOQOL Physical (ΔR^2^ = 0.49, F (4,46) = 13.15, *p* < .001) ○ Posttraumatic growth (*β* = 0.37, 95% CI = [.04, 16.22], *p* < .01) ● WHOQOL Psychological (ΔR^2^ = 0.56, F (4,46) = 17.97, *p* < .001) ○ Posttraumatic growth (*β* = 0.39, 95% CI = [9.18, 16.37], *p* < .001) ▫ Depression (*β* = − 0.31, 95% CI = [9.18, 16.37], *p* < .05) ● WHOQOL Social (ΔR^2^ = 0.34, F (4,46) = 7.51, *p* < .001) ▫ Depression (*β* = − 0.43, 95% CI = [11.21, 21.41], *p* < .05) ● WHOQOL Environmental (ΔR^2^ = 0.38, F (4,46) = 8.79, *p* < .001) ○ Posttraumatic growth (*β* = 0.33, 95% CI = [11.28, 18.86], *p* < .01) ▫ Depression (*β* = − 0.33, 95% CI = [11.28, 18.86], *p* < .05) ▫ Gender (*β* = − 0.26, 95% CI = [11.28, 18.86], *p* < .05) ▫ Unemployment (*β* = − 0.25, 95% CI = [11.28, 18.86], *p* < .05)	Correlations ● Non-significant correlations reported between age and physical, psychological, social, environmental and overall QoL. ● Non-significant correlations reported between gender and physical, psychological, social, environmental and overall QoL. ● Non-significant correlation reported between overall QoL and unemployment Regression model ● Posttraumatic stress symptoms did not significantly predict any of the four domains of QoL ● Gender did not significantly predict physical, psychological or social QoL. ● Depressive symptoms did not significantly predict physical QoL ● Posttraumatic growth did not significantly predict social QoL ● Unemployment did not significantly predict physical, psychological or social QoL
Trilesnik et al. [38]	Germany	Not specified	133	Refugees	Cross-sectional	Psychosocial counseling centers	WHOQOL-BREF	WEMWBS, HSCL-25, HTQ, SCL-90, PMLDC,	● Post-migration living difficulties and overall WHOQOL (*r* = −.54, *p* < .001)	● No significant difference between post-treatment and pre-treatment levels of well-being.
Von Lersner et al. [39]	Germany	Bosnia, Serbia, Kosovo, Iraq, Turkey	100	Refugees (Stayers vs.returnees)	Cross-sectional	Refugee centres, language schools and doctors’ offices.	EUROHIS-QOL	PDS, MINI,	● Stayers ○ Healthy participants vs. those with mental disorder(s) (t (37.4) = 5.65, *p* < .01) with healthy participants having higher QoL ▫ Age and QoL (*r* = −.39, *p* < .05)	● No significant difference in returnees between mentally healthy participants and participants with at least one mental disorder on QoL

^a, b^Same dataset has been used although they addressed different research questions. *Discrepancy exists between how the study used the measure and what the purpose of the measure was intended to be. *AIS* Anger idioms scale. *BDI* Beck Depression Inventory. *BDQ* Brief Disability Questionnaire. *BSI* Brief Symptom Inventory. *CAPS* Clinician Administered PTSD Scale. *CES-D* Self reported depression scale. *CIDI* World Health Organization Composite International Diagnostic Interview. *CRI* Coping Resources Inventory. *CSQ* Culture shock questionnaire. *ETI* Essen Trauma Inventory. *FAD* Family assessment device. *GAD-7* General anxiety disorder. *GAF* General Activity Functioning Assessment Scale. *GHQ-12* General Health Questionnaire. *HDS* Hamilton Depression Scale. *HSCL-25* Hopkins Symptoms Checklist. *HTQ* Harvard Trauma Questionnaire. *ICSEY* International Comparative Study of Ethno-Cultural Youth Questionnaire. *IES-R* Impact of Event Scale-Revised. *ISSI* Interview Schedule of Social Interaction. *LEC* Life Events Checklist. *MINI* International Neuropsychiatric Interview 5.0.0. *MSPSS* Multidimensional scale of perceived social support. *NA* Not Assessed. *PC-PTSD* Primary care PTSD screen. *PCL-C* PTSD checklist civilian version. *PDS* Post traumatic Stress Diagnostic Scale. *PHQ-9* Patient health questionnaire. *PMLP* Post Migration Living Problems. *PTGI-SF* Posttraumatic Growth Inventory Short Form. *SASCAT* Short version of the adapted social capital assessment tool. *SCID-PTSD* Structural Clinical Interview for DSM-IV-TR PTSD Module. *SCL-90* Symptom Checklist. *SDS* Sheehan Disability Scale. *SOC-13* Sense of Coherence Scale. *F-SozU* Social support questionnaire. *WEMWBS* Warwick Edinburgh Mental Well- Being Scale. *WHOQOL-BREF* World Health Organization Quality of Life-Bref. *QLQ* Quality of Life Questionnaire

●Main findings relevant to SWB and/or QoL

⎕Negative Predictor

○Positive Predictor

The WHOQOL-100 is the QoL questionnaire developed by the WHO [40]. It consists of 100 items, and each item is measured from on a 1 to 5 Likert scale. The internal consistency of the Danish version was high (Cronbach’s α = 0.97), with a test-retest reliability of 0.70 [41]. Furthermore, it has been validated in refugee populations [42].

The WHOQOL-BREF is the abbreviated version of the WHOQOL-100 [4] and contains 26 questions. The internal consistency of the WHOQOL-BREF was high (Cronbach’s α = 0.86), and demonstrated discriminant and construct validity (i.e. [43]). It has also been validated in refugee populations [44]. Lastly, the EUROHIS-QOL [45] is an 8-item index which is based on the WHOQOL-100 and WHOQOL-BREF. Each item is measured using the 1 to 5 Likert scale. It has demonstrated high internal consistency (Cronbach’s α = 0.80), and satisfactory convergent and discriminant validity [45].

### Quality of cross-sectional studies

None of the seventeen cross-sectional studies met all 20 quality criteria of the AXIS tool, and although all of the cross-sectional studies met over half of the quality criteria, only 12 met 75% or more of these criteria. The study with the highest quality rating met nineteen of the quality criteria [22] and the study with the lowest quality rating met eleven of the quality criteria [24]. The quality assessment of each cross-sectional study can be found in Table 2.

**Table 2:**
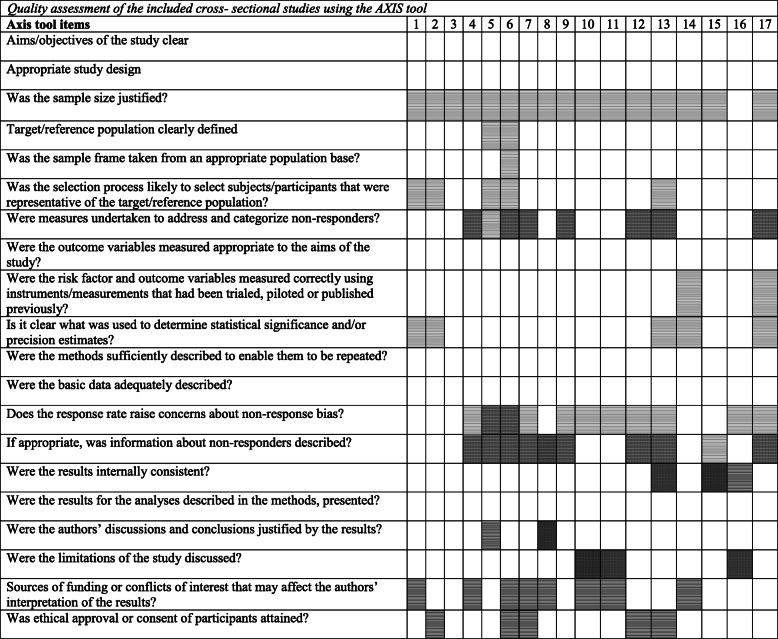
Quality assessment of the included cross- sectional studies using the AXIS tool

1 = Carlsson et al. [19], 2 = Lee et al. [31], 3 = Correa-Velez et al. [22], 4 = Correa-Velez et al. [21], 5 = Georgiadou [23], 6 = Ghazinour et al. [24], 7 = Hengst et al. [25], 8 = Huijts et al. [26], 9 = Jesuthasan et al. [27], 10 = Laban et al. [29], 11 = Laban et al. [30], 12 = Leiler et al. [32], 13 = Regev et al. [34], 14 = Slonim-Nevo et al. [35], 15 = Teodorescu et al. [37], 16 = Trilesnik et al. [38], 17 = Von Lersner et al. [39]

Quality met

Quality not met

Unclear

Many methodological weaknesses were noted. Firstly, sample size justification (i.e. power calculation) was only reported by one study [38]. Five studies were unclear regarding sample selection, and one study was unclear regarding taking the sample frame from an appropriate population base [24]. Secondly, there were significant concerns regarding response bias as eight studies did not make a clear attempt to quantify the level of non-responders. Thirdly, five studies were unclear on standards used for determining statistical significance and/or precision estimates in their results section. This was due to insufficient detail regarding data management, significance levels, effect sizes and/or confidence intervals. Lastly, eight studies did not clearly report sources of funding and/or conflicts of interest. Five studies were not clear on whether ethical approval or consent had been obtained.

### Quality of longitudinal studies

Table 3 provides details about the quality of the longitudinal studies. Five studies clearly defined their primary outcome, one did not [28]. Five also used validated measures, one did not [18]. All of the studies identified confounding factors, however two did not take them into account in the analysis, and two studies were unclear. Overall, a range of follow-up periods were used; 6-months, 7-months, 9-months, 12-months, 14-months, 23-months and 10-years.

**Table 3:** Quality Assessment of the Included Longitudinal Studies using the CASP Tool

CASP Tool	Carlsson et al. *Baseline* vs. *9-month follow-up* [18]	Carlsson, Olsen, Mortensen & Kastrup *10-year follow-up* [20]	Carlsson et al. *Baseline* vs *9 month* vs. *23 month follow-up* [17]	Kinzie et al. *Baseline* vs*- 12 month follow-up* [28]	Löfvander et al. *Baseline, 6- and 12-month follow-up* [33]	Stammel et al. *Baselinve* vs. *7 months* vs. *14 months* [36]
Did the study address a clearly focused issue?	Yes	Yes	Yes	No	Yes	Yes
Was the cohort recruited in an acceptable way?	No	Yes	Yes	Yes	Yes	Yes
Was the exposure accurately measured to minimise bias?	Cannot tell – no control group	Cannot tell – no control group	Yes	Cannot tell – no control group	Yes	Cannot tell – no control group
Was the outcome accurately measured to minimise bias?	Yes	Yes	Yes	Yes	Yes	Yes
Have the authors identified all important confounding factors?	Yes	Yes	No	Yes	Yes	Yes
Have they taken account of the confounding factors in the design and/or analysis?	No	No	Cannot tell	Cannot tell	Yes	Yes
Was the follow up of subjects complete enough?	Yes	Yes	Yes	Yes	Cannot tell	Cannot tell
What are the results of this study?	After a mean of 8 months of multidisciplinary treatment, mental symptoms and health-related quality of life did not change	The level of emotional distress was high at follow-up. Social relations and unemployment at follow-up were important predictors of mental health symptoms and low health-related quality of life.	Reduction in trauma /depression (baseline > 23 month) means. Minimal differences due to low effect sizes. Intervention not effective.	There were significant changes between means on the WHOQOL physical, mental and environmental domains after 1 year.	New immigrants did not have inferior physical or psychological health, quality-of-life, well-being or social functioning compared with their age- and sex-matched Swedish born pairs during a 1-year follow-up.	Quality of life increased significantly after an average of 14 months of treatment.
How precise are the results?	Cannot tell	Cannot tell	Cannot tell	Cannot tell	Cannot tell	Good
Do you believe the results?	Cannot tell	Cannot tell	Yes	No, more information is required	Cannot tell	Yes
Can the results be applied to the local population?	No	No	No	No	No	No
Do the results of this study fit with other available evidence?	Yes	Yes	Cannot tell	Cannot tell	No	No
What are the implications of this study for practice?	When planning health-related and social interventions an increased focus is needed on the present exile situation, e.g., social relations, occupation and resources available in the present situation.	Post migratory factors, such as social relations and occupation, are important for mental health and health-related quality of life. For the clinician dealing with severely traumatized refugees, it is important to be aware of a possible chronic condition.	Long-term follow-ups should be included in randomized trials focusing on the effects of different treatment approaches, including the appropriate length of treatment.	The results can have implications for the treatment of torture survivors.	General screening in unselected settings of refugees and new immigrants seems to be of little value. Clinical consultations in selected cases are to be preferred, adopting a holistic practical approach in patient and family-focused care.	It provides evidence for the efficacy of multidisciplinary treatment, more research needed.

The two biggest limitations were that all studies lacked statistical precision (e.g. failing to state the confidence intervals or effect size), and the results cannot be applied to the local population as studies were conducted on very specific samples. There was a shortage of detail regarding follow-up assessments – three studies did not provide enough information on non-responders [18, 20, 33]. All but one [36] study did not clearly report effect sizes, variance accounted for by regression models, and/or the confidence intervals for the results. The quality assessment of each individual longitudinal study can be found in Table 3.

### Overall quality of life (oQoL)

All WHOQOL-BREF domains positively correlated with each other [37]. There was evidence of differences in oQoL according to the time that had passed since arriving in the host country – Two studies, using the same sample, found that asylum seekers who had recently resettled (< 6 months) rated their oQoL higher than those who had lived in the host country for at least 2 years [29, 30]. Simultaneously, Stammel et al. found that refugees’ oQoL increased after 14 months of multidisciplinary treatment.

In terms of *physical* correlates, significant gender differences were found - males reported lower oQoL than females [24], and Regev et al. [34] found gender to be a significant negative predictor of oQoL, however the coding of variables was not reported. When compared to a non-refugee EU sample, a female refugee sample rated their oQoL significantly lower [27]. Being older (> 30 years) predicted lower oQoL [27], and was a negative correlate of oQoL [39].

*Psychological* associations with lower oQoL included, self-rated PTSD [26], posttraumatic stress [37], depression [23, 24, 37] and having one or more mental disorders, including depression, anxiety, PTSD and somatoform disorders [25]. Furthermore, experiencing the following adverse events was negatively associated with oQoL; near-death experiences [27], self-traumatic events [34], forced isolation [27], adverse events post-resettlement [30], and other traumatic experiences [25, 34].

When compared to individuals with a mental disorder, healthy individuals reported higher oQoL [39]. Sense of coherence was positively associated with oQoL [23, 24], with males reporting a significantly lower sense of coherence than females [24]. Exposure to other people’s traumatic events [34] and posttraumatic growth [37] were positive predictors of oQoL; and coping strategies [24, 26], availability and adequacy of attachment [24] correlated with increased oQoL. According to one study, coping strategies only led to an increase in oQoL for females [26]. Exposure to other people’s traumas was interpreted by the authors as potentially providing validation for people’s own experiences [34].

Weak social networks and poor social integration were *social* correlates of low oQoL [37]. Specific events that predicted lower oQoL included the unnatural loss of a child [25], attacks by family members^2^ [27], and number of lost family members [25].

Positive *social* predictors of oQoL focused on having social support [23, 26, 34]. Additionally, social integration [24] and having a spouse in the host country [23, 27], were associated with higher oQoL. One study suggested that social support was only a significant predictor for males [26].

Three of the *environmental* predictors that were investigated predicted low oQoL; prior mission/residence in a war zone [27], being sick without access to healthcare and long asylum procedures [25, 30]. Post-migration living difficulties negatively correlated with oQoL [38]. Similarly, one study found that North Korean refugees resettled in South Korea vs. those resettled in Japan had higher QoL, which the authors interpreted as being due to difficulties adapting to a new culture [31]. No positive predictors or correlates of eQoL were found.

**Fig. 2:**
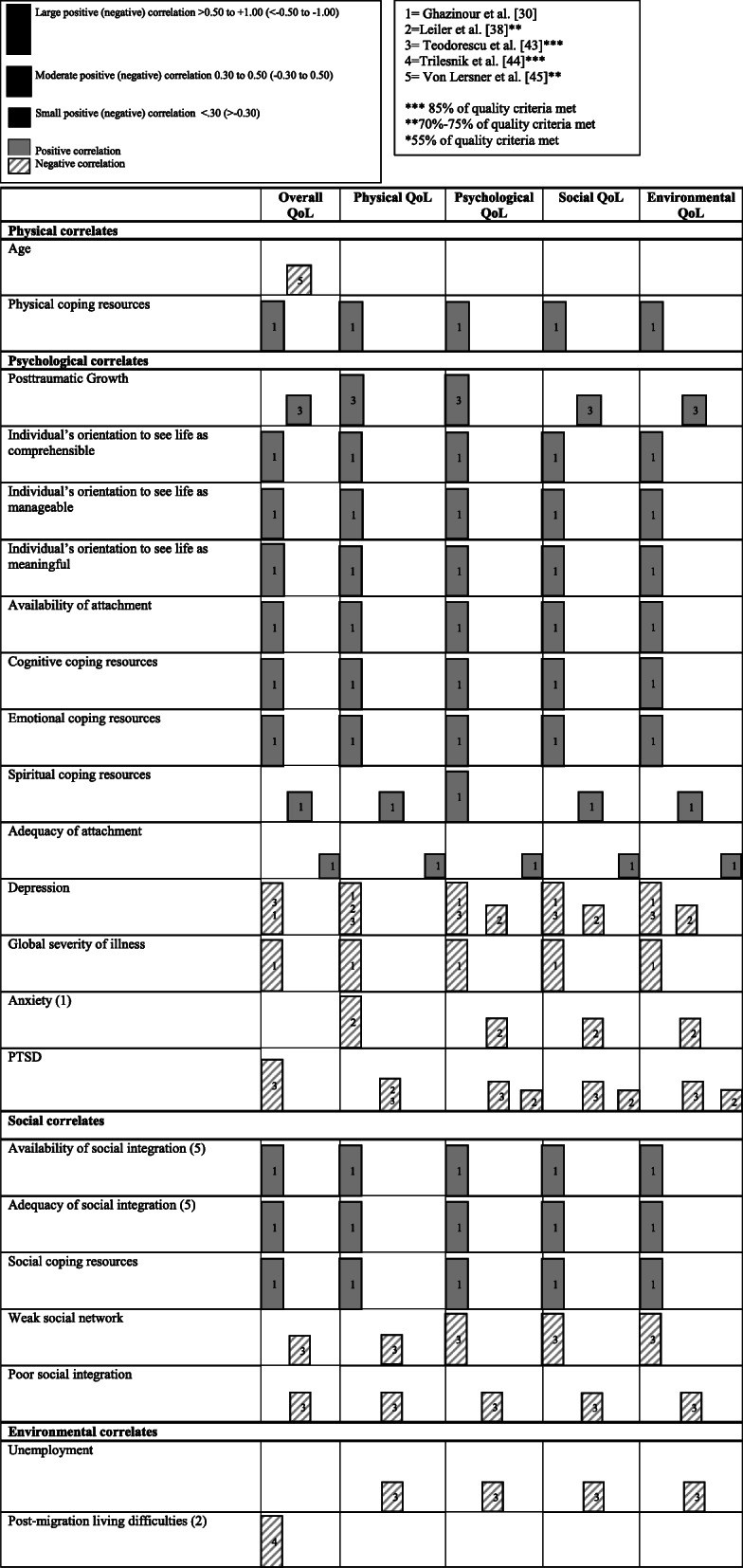
A harvest plot indicating the positive and negative correlations of overall QoL and the four QoL domains. All findings are from cross-sectional studies

The consideration of the direction, strength and consistency of the correlational analyses of the correlates of oQoL reported across studies is summarized in Fig. 2. For oQoL, both the strongest positive and negative correlations were found by Ghazinour et al. The strongest positive correlate found was between physical coping resources and psyQoL (*r* = 0.82, *p* < .001) and the strongest negative correlation found was between depression and psyQoL (*r* = − 0.86, *p* < .001) [24]. However, this study reported the lowest quality of the 23 studies included. The majority of strong positive correlations found for oQoL were *mental correlates.*

### Physical quality of life (pQoL)

Laban et al. [30] found that asylum seekers who had recently resettled (< 6 months) rated their pQoL higher than those who had lived in the host country for at least 2 years [30]. On the other hand, Kinzie et al. [28] reported pQoL improved over time (1 year) for refugees who were undergoing treatment. Older age was a negative predictor of pQoL [30]. Negative *Physical* correlates of pQoL in AS&R were physical pain [19] and headaches [20]. The only positive *Physical* predictor found was region of birth, specifically being African was a positive predictor of pQoL [21].

Negative *Psychological* predictors of pQoL included diagnoses of depression [30], somatoform disorders [30], PTSD [35], having one or more mental disorders [30], and adverse life events post-migration [30]. Negative correlates of pQoL found were similar; depression [24, 32, 37], anxiety [32] and PTSD [32, 37]. Contrarily, coping strategies [24], availability and adequacy of attachment [24], were correlated with increased pQoL [37]. Posttraumatic growth was a positive predictor of pQoL [37].

One study reported on negative *social* predictors of pQoL and found that perceived discrimination [35] negatively predicted pQoL. Weak social networks [37] and poor social integration [37] were negatively correlated with pQoL. Positive *social* predictors were having social relations [19, 20] and feeling that most people in the community can be trusted [21]. Additionally, social integration was positively correlated with pQoL [24].

Living conditions post-resettlement, specifically socio-economic conditions [30] was a significant negative *environmental* predictor of pQoL. Unemployment was significantly negatively correlated with pQoL but was not a significant predictor [37]. Being employed [19, 20] and having completed either secondary or tertiary education [21] were significant *environmental* predictors of increased pQoL [19, 20]. Additionally, legal status increased pQoL, with refugees reporting higher pQoL than asylum seekers [35]. Lastly, place of resettlement was significant, as one study found that North Korean refugees resettled in South Korea vs. those resettled in Japan had higher QoL, which the authors interpreted as being due to difficulties adapting to a new culture [31].

### Psychological quality of life (psyQoL)

Differing results were found for asylum seekers and refugees on psyQoL over time. Laban et al. [30] found that asylum seekers who had recently resettled (< 6 months) rated their pQoL higher than those who had lived in the host country for at least 2 years [30], whereas Kinzie et al. [28] reported psyQoL improved over time (1-year) for refugees who were undergoing multidisciplinary treatment [28]. Group comparisons over time between refugees and a non-migrant sample also showed that refugees scored significantly higher on psyQoL outcomes at baseline, 6 months and 12 months [33].

The only *physical* predictor for psyQoL was gender, with males reporting higher psyQoL overall [23]. Males also reported lower levels of independence and spirituality than females, which belong to the psychological domain [24].

*Psychological* predictors found to decrease psyQoL were depression [23, 37], anxiety [36,], PTSD [30, 35], and somatoform disorders [30]. Negative correlates for psyQoL found were similar; depression [24, 32, 37], anxiety [32] and PTSD [32, 37].

*Psychological* correlates for an increased psyQoL were self-evaluations of improved MH during treatment [18] coping strategies [24], and availability and adequacy of attachment [24]. Sense of coherence and posttraumatic growth [37] positively predicted psyQoL [23].

Negative *social* predictors reported were perceived discrimination [35], and number of key persons who provide no support [21]. Having a weak social network and poor social integration negatively correlated with psyQoL [37]. Findings on positive *social* predictors relevant to psyQoL highlighted the importance of social support [19, 20, 23], feeling that most people in the community can be trusted [21], and having one’s spouse in the host country [23]. Additionally, social integration [24] positively correlated with psyQoL.

The only significant negative *environmental* predictor of psyQoL was poor socio-economic living conditions post-resettlement [30]. Unemployment was a negative correlate [37]. Lastly, place of resettlement was significant as one study found that North Korean refugees resettled in South Korea vs. those resettled in Japan had higher QoL [31].

### Social quality of life (sQoL)

There were two *physical* correlates of sQoL - having headaches predicted lower sQoL [20], and Gender. Löfvander et al. [33], noted that whereas the male refugees had higher sQoL compared to Swedish born controls (matched for age and gender) at the three assessment points (baseline, 6 months and 12-month follow-up), female refugees had significantly higher sQoL relative to Swedish born matched controls at baseline assessment only. Another study reported males had lower sQoL than female refugees [24].

Negative *psychological* correlates of sQoL reported were; depression [24, 32, 37], anxiety [32], PTSD [32], and post-traumatic stress [37]. PTSD [35] and depression [37] were negative predictors of sQoL. Availability and adequacy of attachment [24], and coping strategies [24] were positively correlated with sQoL, and posttraumatic growth positively predicted sQoL [37]. Perceived discrimination [35], was a significant negative *social* predictor of sQoL. Simultaneously, weak social network [37], and poor social integration [37] were negatively correlated with sQoL. Social integration [24] and being married with a spouse in the host country [23] were positively correlated with sQoL, and social relations [19, 20], positively predicted sQoL.

Employment was the only *environmental* predictor found to increase sQoL [20], and unemployment was found to decrease sQoL [37] Additionally, one study found that North Korean refugees resettled in South Korea vs. those resettled in Japan had higher QoL, which the authors interpreted as being due to difficulties adapting to a new culture [31].

### Environmental quality of life (eQoL)

Three studies revealed the eQoL increased over time for refugees, after 9-months [17], 12-months [28] and 23-month follow-up [17].

Laban et al. [30] found that asylum seekers who had recently resettled (< 6 months) rated their eQoL higher than those who had lived in the host country for at least 2 years [30].

In terms of negative *physical* predictors of eQoL, studies reported on the presence of pain [19] and headache [20]. Gender was also a predictor of eQoL; however, the authors did not specify how gender was coded [37]. However, Ghazinour et al. [24], found that males reported lower eQoL than females.

The negative *psychological* predictors of eQoL were self-rated PTSD [30], and depression [37]. Negative correlates found were similar; depression [24, 32], anxiety [32] and PTSD [32] negatively correlated with pQoL. Positive *psychological* correlates of eQoL were coping strategies [24], availability and adequacy of attachment [24]. Posttraumatic growth was a positive predictor of eQoL [37].

Perceived discrimination [35], was a significant negative predictor of eQOL. Poor social integration and having a weak social network [37] were negatively correlated with eQOL. The significant positive *social* predictor identified for eQOL was having social relations [19, 20]. Social integration [24] positively correlated with eQoL and having one’s spouse in the host country was associated with higher eQoL, as compared to not having one’s partner in the host country [23].

The negative *environmental* predictors found comprised socio-economic living conditions post resettlement (including living in regional areas as opposed to central areas) [22, 30], post-migration living difficulties [35], unemployment [37], and socio-religious aspects, such as a lack of contact with people of the same religion [30]. The significant positive *environmental* predictor of eQoL was being employed [20]. Additionally, place of resettlement was found to be significant in two studies; Correa-Velez et al. [22] found that living in regional areas was a positive predictor of eQoL, and Lee et al. [31] found that North Korean refugees resettled in South Korea vs. those resettled in Japan had higher QoL.

### Differences between asylum seekers and refugees

Sixteen of the included studies focused on refugees, three on asylum seekers and four used mixed samples or terminology. Only nine studies (39.1%) gave the specific criteria used to define their sample as either a refugee or asylum-seeking population (i.e. by law). This is important as some studies used mixed terminologies or did not distinguish between the two. Given that asylum seekers and refugees constitute different populations with different needs, this distinction is important. Only two studies specifically compared asylum seekers to refugees [32, 35]. Leiler et al. [32] found no significant differences between them on any of the four QoL domains nor in oQoL. Slonim-Nevo [35] did find that having a legal status positively predicted pQoL. Similarly, long asylum procedures were found to be a negative predictor for oQoL by all three studies that focused exclusively on asylum seekers [25, 29, 30]. However, Laban et al. [30] did not find long asylum procedures to be a significant predictor for pQoL specifically. Furthermore, for asylum seekers, QoL did not appear to improve over time whereas for refugees findings suggest that it does.

## Discussion

To date, there has been a paucity of efforts to synthesize evidence relating to predictors and correlates of QoL of AS&Rs. The current review sought to address this gap, so that policy makers and organizations working to support AS&Rs in high-income countries can be guided by an improved understanding about what enhances the lived experience of AS&Rs. Key findings across the various forms of QoL (overall, physical, psychological, social and environmental) were that having established social networks and social integration were associated with higher QoL, whereas having mental disorders (i.e. PTSD or depression) was strongly associated with reduced QoL. Physical predictors and correlates were the least reported.

Psychological predictors and correlates (including the presence of mental disorders) of QoL were the most extensively studied and reported across studies. The predictors and correlates of QoL noted in the current review can be compared with predictors of common mental disorders (CMD) identified in previous reviews. For example, Bogic et al. [46] found that poor post-migration socio-economic status including unemployment, low income, poor host language proficiency and lack of social support were each associated with depression experienced by war-affected refugees. These findings overlap with those found in the current review. However, there were also important points of distinction; the current review showed that having a spouse was positively associated with increased QoL, whereas Bogic et al. [46] did not find any consistent association between marital status and mental disorders. Furthermore, this review showed that positive coping strategies were highly associated with increased QoL, whereas Bogic et al. [46] indicated that these factors had not been assessed in studies exploring mental disorders experienced by war-affected refugees. To ensure that AS&Rs are afforded the opportunity to enjoy full and meaningful lives, it will be important to understand and address not only factors associated with mental disorders, but also those uniquely associated with QoL.

The associations that QoL had with various social factors and environmental factors, point to the value of extensive integration programs that include housing and employment assistance [47]. Unfortunately, however, in many high-income countries, AS&Rs face social exclusion, restricted employment opportunities, and/or below average earnings [48]. The current review highlighted that having weak social networks, and poor social integration were both moderately correlated with lower overall QoL. Those involved in developing migrant integration policies need to be cognizant of the associations that QoL have with various aspects of the socio-ecological context that AS&Rs live in. Most European governments and other OECD countries outside Europe have imposed employment bans or time constraints to asylum seekers entering the labor market [49, 50]. Although asylum policies vary by country, region, and even over time within a country, such policies generally lead to long waiting periods in which asylum seekers find themselves in a legal and social limbo, without the ability to work and integrate. Research has shown that longer waiting times to obtain a refugee status strongly reduces employment integration of refugees (i.e. [51–53]) and can also reduce social integration (i.e. [54, 55]). Therefore, the findings of the current review should be considered by policymakers as being consistent with a need to reduce asylum procedure times, in order to promote socio-economic integration, reduce the risk of marginalization and mental ill-health, and overall increase the QoL of AS&R populations. The discourse must shift from a narrative regarding AS&R as being a burden on society to seeing their support as an investment in the social and economic framework of the host country.

Regarding methodological quality, studies had moderate to good quality overall with more recent publications generally scoring higher on quality assessment. There was a tendency to recruit opportunistic samples through health centers, which may have resulted in a bias towards AS&R who were already seeking care and with greater support systems rather than more marginalized individuals. This limits the generalizability of findings. Evidence of basic design flaws, and the predominance of cross-sectional methodologies were important limitations of the available evidence base. Moving forward more transparency is required regarding sampling procedures and non-responders. Furthermore, authors need to clearly state sources of funding and possible conflicts of interest that may have led to outcome bias.

### Future research and implications

The current review has highlighted a need for research to further explore factors positively associated with QoL. Mixed-method approaches may be used to allow for a qualitative exploration of context and culture, together with a quantitative prediction. Longitudinal studies aimed at exploring causal relationships that variables (including mental disorders) potentially have with QoL are required. Specifically more research is needed on environmental and physical correlates and predictors of QoL. Clinical trials of interventions conducted with AS&R populations that employ instruments assessing QoL as primary outcomes is required. This is a particularly worthy area of research focus in light of the fact that many people opt not to engage with treatments for mental disorders owing to the stigma that it can bring [56].

### Limitations of this review

The exclusion of grey literature may have introduced a publication bias into the findings presented in the current review. However, the peer review process for journal submission was used as a form of minimal quality assurance for the studies included in the review.

Similarly, the exclusion of articles that were not written in a language spoken by the authors (English, Spanish or Dutch) may have introduced a language bias. These decisions were made due to authors’ language proficiency and a lack of time to arrange translating resources. Therefore, publications on other languages should be considered an area for future research.

The samples of the included studies varied significantly with respect to country of origin, time since resettlement, year and country of study publication. However, this reflects the reality that AS&Rs populations tend to be very diverse in terms of their personal circumstances. Furthermore, studies recruiting AS&Rs in low- and middle-income countries were excluded given that this review aimed to support efforts to provide further evidence to guide health and social care policy that could inform the support of AS&R in high-income countries. As the majority of the world’s AS&R live in low- and middle- income countries [1], this limitation highlights the importance of further research concerning the factors influencing AS&Rs’ QoL in low and middle-income settings. The analyses used in the studies included in the current review do not permit causal relationships to be inferred. Finally, there was heterogeneity in the measures of QoL used and this limited efforts to synthesize the findings. Consideration was also given to conducting a meta-analysis but given the large heterogeneity of available data, a meta-analysis was deemed inappropriate.

## Conclusion

In summary, this review expands knowledge on the predictors and correlates of QoL in AS&R populations. The findings highlight that there are significant physical, psychological, social and environmental predictors and correlates that affect QoL in AS&Rs. Overall, the majority of strong positive correlations found for oQoL were MH related correlates*.* Positive MH is a key determinant for good integration [57, 58], and good integration is a determinant of good MH [59]. Efforts to develop and deliver interventions to support AS&Rs need to be aware of QoL as an important outcome and target important determinants thereof.

### Supplementary information

**Additional file 1.** Appendix B Positive and negative predictors of overall QoL and each of the four domains.

## Data Availability

The datasets used and/or analysed during the current study are available from the corresponding author on reasonable request.

## Appendix

Database Search Strategy

The databases searched were Medline, PsycINFO, CINAHL, Cochrane Library, Health Technology Assessment, National Health Service Economic Evaluation, Educational Resource Index and Abstracts, BiblioMap, Scopus, Social Sciences Citation Index, Evidence Aid, DARE, Web of Science and PubMed) up to 5th of May, 2020. The Scopus database enabled searching Embase articles not indexed in the previous databases.

Search Strategy.

**Table:**

Concept	Keywords	Controlled Vocabulary	Example database Psychinfo
• Asylum seekers	Asylum seeker	Asylum Seeker	• Asylum Seekers OR Refugees OR political asylum OR political refugee
• Refugees	Refugee	Seekers, Asylum	• asylum N2 seek* OR refuge* OR
• Political asylum	Political asylum	Refugee	Seeker*, Asylum OR political N2 asylum OR political N2 refuge*
• Political refugees	Political refugees	Stateless people	
• Adults	Adults	Political asylum	• Adult*
		Political refugees	
		Adult	
• Quality of Life	Wellbeing	Wellbeing	·Wellbeing OR Subjective wellbeing OR Psychological wellbeing OR Emotional wellbeing OR Quality of life OR QoL OR Life quality OR Health-related quality of life OR Life satisfaction OR Satisfaction with life
• Wellbeing	Subjective wellbeing	Subjective wellbeing	
	Psychological wellbeing	Psychological wellbeing	
	Emotional wellbeing	Emotional wellbeing	
	Quality of life	Quality of life	·Well N2 being OR Subjective N3 well N2 being OR Psychological N3 well N2 being OR Emotional N3 well N2 being OR Quality N3 of N3 life OR QoL OR Life N2 quality OR Health N2 related N5 quality N3 of N3 life
	Life quality	QoL	
	Life satisfaction	Life quality	
		Health-related quality of life	
		Life satisfaction	OR Life N3 satisfaction
		Satisfaction with life	
• Predictive Terms	Predictor	Predictor	· predictor OR correlation OR determinant
	Correlation	Correlation Determinant	·predict* OR correlat*
	Determinant		OR determin*

## Footnotes

1 Further information on the classification of countries per income can be found at: https://datahelpdesk.worldbank.org/knowledgebase/articles/906519

2 This terminology was replicated from the study itself, the authors of the study do not specify what is meant specifically with this term.
