# Supplementary material for: Systematic review of factors associated with quality of life of asylum seekers and refugees in high-income countries
Source: Confl Health. 2020 Jul 20;14:48. doi: 10.1186/s13031-020-00292-y (PMC7370437; doi:10.1186/s13031-020-00292-y)
Supplement: Supplementary file 1 — Additional file 1. Appendix B Positive and negative predictors of overall QoL and each of the four domains. [file 13031_2020_292_MOESM1_ESM.docx]

**Appendix B - Positive and negative predictors of overall QoL and each of the four domains.**

Positive predictor

Negative predictor

Self-traumatic events

Social support

Other’s traumatic events

Unnatural loss of a child

Social predictors

-0.20***[40]

Adverse events post-migration

0.32***[40]

Nº of lost family members

-0.05*[31]

0.27***[40]

-0.13*[36]

-0.04*[31]

Emotion focused coping

0.13**[32]

0.15*[29]

-0.18*[31]

Sense of coherence

Psychological predictors

-0.44***[29]

-0.13*[31]

Long asylum procedure

-0.18*[31]

Depression

-0.61***[32]

Other traumatic events

Environmental predictors

Psychopathology

Psychopathology

*Gender and time have not been included in the figure. Where multiple studies reported the same predictor only the study with the strongest predictor is reported in the figure.

***p<001, **p<.01, *p<.05

-0.19* [26]

-0.35* [25]

-0.38*** [41]

0.40*** [26]

-0.14*[36]

0.20* [27]

-0.12*[36]

-0.30*** [41]

-0.31*** [26]

-0.14**[36]

*Gender and time have not been included in the figure. Where multiple studies reported the same predictor only the study with the strongest predictor is reported in the figure.

***p<001, **p<.01, *p<.05

0.40*** [26]

-0.25*[43]

0.12*[36]

Unemployment

Socio-religious aspects

Living difficulties post-migration

Socio-economic living conditions

Employment

Education

Legal status

Discrimination

Residence of partner

No support

Social support

Environmental predictors

Social predictors

0.49*** [26]

-0.15* [41]

0.40** [25]

Community can be trusted

Psychological predictors

Physical predictors

PTSD

PTGrowth

Somatoform disorders

Anxiety

Depression

Sense of coherence

Adverse events post-migration

Mental disorders

Older age

Headache

Pain

Region of birth

-0.24** [41]

0.17* [29]

-0.24* [27]

0.31* [25]

0.33** [25]

0.20* [27]

0.14**[41]

-0.20**[36]

0.32** [27]

0.36*** [26]

-0.38***[41]

-0.27**[36]

0.32*** [27]

-0.42** [25]

-0.19*[36]

-0.15*[36]

0.22**[29]

-0.20* [26]

0.37**[43]

-0.19**[36]

-0.40*** [29]

-0.43*[43]

-0.17*[36]

-0.15**[36]

0.39***[43]

0.33**[43]

-0.14*[36]

-0.27***[41]

-0.29***[41]

-0.33*[43]

0.27** [26]
